# Phylogenomics of Cas4 family nucleases

**DOI:** 10.1186/s12862-017-1081-1

**Published:** 2017-11-28

**Authors:** Sanjarbek Hudaiberdiev, Sergey Shmakov, Yuri I. Wolf, Michael P. Terns, Kira S. Makarova, Eugene V. Koonin

**Affiliations:** 1 National Center for Biotechnology Information, National Institutes of Health, Bethesda, MD USA; 2 Skolkovo Institute of Science and Technology, Skolkovo, 143025 Russia; 3 Departments of Biochemistry and Molecular Biology, Genetics, and Microbiology, University of Georgia, Athens, GA USA

## Abstract

**Background:**

The Cas4 family endonuclease is a component of the adaptation module in many variants of CRISPR-Cas adaptive immunity systems. Unlike most of the other Cas proteins, Cas4 is often encoded outside CRISPR-*cas* loci (solo-Cas4) and is also found in mobile genetic elements (MGE-Cas4).

**Results:**

As part of our ongoing investigation of CRISPR-Cas evolution, we explored the phylogenomics of the Cas4 family. About 90% of the archaeal genomes encode Cas4 compared to only about 20% of the bacterial genomes. Many archaea encode both the CRISPR-associated form (CAS-Cas4) and solo-Cas4, whereas in bacteria, this combination is extremely rare. The solo-*cas4* genes are over-represented in environmental bacteria and archaea with small genomes that typically lack CRISPR-Cas, suggesting that Cas4 could perform uncharacterized defense or repair functions in these microbes. Phylogenomic analysis indicates that both the CRISPR-associated *cas4* genes are often transferred horizontally but almost exclusively, as part of the adaptation module. The evolutionary integrity of the adaptation module sharply contrasts the rampant shuffling of CRISPR-*cas* modules whereby a given variant of the adaptation module can combine with virtually any effector module. The solo-*cas4* genes evolve primarily via vertical inheritance and are subject only to occasional horizontal transfer. The selection pressure on *cas4* genes does not substantially differ between CAS-Cas4 and solo-*cas4*, and is close to the genomic median. Thus, *cas4* genes, similarly to *cas1* and *cas2*, evolve similarly to ‘regular’ microbial genes involved in various cellular functions, showing no evidence of direct involvement in virus-host arms races. A notable feature of the Cas4 family evolution is the frequent recruitment of *cas4* genes by various mobile genetic elements (MGE), particularly, archaeal viruses. The functions of Cas4 in these elements are unknown and potentially might involve anti-defense roles.

**Conclusions:**

Unlike most of the other Cas proteins, Cas4 family members are as often encoded by stand-alone genes as they are incorporated in CRISPR-Cas systems. In addition, *cas4* genes were repeatedly recruited by MGE, perhaps, for anti-defense functions. Experimental characterization of the solo and MGE-encoded Cas4 nucleases is expected to reveal currently uncharacterized defense and anti-defense systems and their interactions with CRISPR-Cas systems.

**Electronic supplementary material:**

The online version of this article (10.1186/s12862-017-1081-1) contains supplementary material, which is available to authorized users.

## Background

Cas4 is one of the core CRISPR-associated (Cas) proteins, which is implicated in the adaptation phase in several subtypes of CRISPR-Cas systems [1–4]. During this phase of the CRISPR response, a segment of invading DNA (protospacer), usually from a virus or plasmid, is selected, typically, based on the presence of a protospacer adjacent motif (PAM) [5, 6]. The protospacer then is incorporated by the Cas1-Cas2 adaptation complex into a CRISPR array, most often, next to the first, 5′-terminal repeat. The array is transcribed into a single, long pre-crRNA, which is subsequently processed into mature CRISPR (cr)RNAs and is incorporated into the effector complex or a single effector protein, which scans invading DNA and, once a match is found, recruits nuclease domain-containing Cas proteins to cleave the target DNA [7–10]. So far the *cas4* gene has been identified in the subtypes I-A, B, C, D, U of Class 1 CRISPR-Cas systems and subtypes II-B and V-A, B, E of Class 2. In each of the respective loci, the *cas4* gene is adjacent to *cas1* and *cas2*, and in some cases, fused with *cas1*, which is compatible with the involvement of Cas4 in adaptation [11, 12]. Furthermore, it has been shown that in subtype I-A loci, *cas1*, *cas2* and *cas4* genes form a single operon [1], and for subtype I-B, the requirement of Cas4 for adaptation has been demonstrated in a direct experiment [3]. A recent study on the cas gene requirement for adaptation by the type I-A system of the crenarchaeon *Sulfolobus islandicus* has shown that two paralogous *cas4* genes (one denoted *csa1*) in the *cas* operon are both essential, suggesting that these proteins could form a complex in which the two paralogs would perform unique functions [13].

Cas4 is a DNA exonuclease that contains a nuclease domain and a Fe-S cluster-binding module [2, 14–16] and is homologous to the nuclease moieties of well-characterized proteins involved in recombination and repair in bacteria (RecB, AddB) and eukaryotes (Dna2) [17–21]. The Cas4 nuclease belongs to the expansive PD-DEXK phosphodiesterase superfamily, named after the conserved catalytic motif present in most members [21]. Similar to the AddB nuclease, Cas4 is thought to form recombinogenic 3′ ssDNA overhangs in the protospacers, thus facilitating their subsequent incorporation into the CRISPR array as dsDNA spacers [16]. In the structures that have been solved for Cas4 proteins from *Pyrobaculum calidifontis* (Pcal_0546; pdb: 4R5Q) [15] and *Sulfolobus solfataricus* (SSO0001, pdb: 4IC1) [2], Pcal_0546 is a monomer, whereas SSO0001 forms a toroidal decamer composed of 5 tightly packed dimers. The Pcal_0546 protein has been shown to exhibit a metal-dependent 5′ to 3′ exonuclease activity against ssDNA substrates [15], whereas the Cas4 protein SSO1391 from *S. solfataricus* has been reported to cleave ssDNA in both the 5′ to 3′ and the 3′ to 5′ directions [2]. However, in an independent study, only the 5′-3′ exonuclease activity has been detected for SSO1391 as well [16].

Although the *cas4* gene is usually located next to *cas1* or *cas2* genes and, in some CRISPR-Cas systems, is fused to *cas1* (eg. subtypes I-U and V-B), many bacterial and archaeal genomes encompass an additional *cas4* gene that is not associated with CRISPR-*cas* loci (hereinafter, solo-Cas4). Notably, solo-cas4 genes are present in many archaeal and bacterial genomes that lack CRISPR-*cas* loci [11].

The recent discovery of Cas4 homologs encoded in bacteriophage genomes has added a new twist to the functional repertoire of the Cas4 family. It has been shown that a *Campylobacter* phage that encodes a Cas4-like protein, employs this protein to stimulate acquisition of host-derived spacers by the *Campylobacter* type II-C CRISPR-Cas system (which lacks *cas4*). The phage appears to use this mechanism to evade host immunity via a mechanism that remains to be characterized [22, 23].

Cas4 homologs have been identified also in archaeal viruses [24] and casposons, self-synthesizing transposons that employ Cas1 homologs as recombinases and are thought to be the ancestors of the CRISPR-Cas adaptation modules [25, 26]. One archaeal virus, Thermoproteus tenax virus 1 (TTV1), encodes an inactivated derivative of a Cas4-like nuclease that became a component of the viral nucleocapsid [24].

Despite the current keen interest in the mechanisms, diversity and evolution of the CRISPR-Cas systems, no comprehensive analysis of the Cas4 family has been reported so far. The aim of this work is to present such an analysis with the focus on *cas4* gene neighborhoods and comparison of the (predicted) functions and evolutionary regimes of CRISPR-associated Cas4 and solo-Cas4 subfamilies.

## Results and discussion

### Phylogenomic analysis of the Cas4 protein family

As reported previously, the majority of the Cas4 proteins belong to two families, namely, COG1468 and COG4343; the latter is specific for subtype I-A CRISPR-Cas systems and is also known as Csa1 (these two COGs correspond to pfam01930/DUF83 and pfam06023/ DUF911, respectively) [11]. In addition, Cas4-like nucleases of pfam12705/PDDEXK_1 are occasionally found in association with *cas* loci and have been shown to affect spacer adaptation by certain CRISPR-Cas systems [22, 23]. In the present analysis, we focused primarily on these three major families of Cas4 homologs and disregarded a few other, less common PD-DExK families that also have been reported in the vicinity of several CRISPR-*cas* loci [11].

To collect protein sequences of the Cas4 family, we screened 48,599 prokaryotic genomes (Genbank, March 2016) using PSI-BLAST search [27] with the sequence profiles related to the three families of Cas4 homologs. The results of this search were filtered to exclude sequences that produced better scores to other profiles in the CDD database (See Material and Methods for details). Additionally, we searched 2996 phage genomes from the PHANTOM database [28]. In total, 7060 protein sequences were retrieved, of which 883 were from complete bacterial and archaeal genomes, and 272 were from bacterial or archaeal viruses.

Inspection of the multiple alignment of Cas4 proteins (see Materials and Methods) shows conservation of the catalytic motifs of the PD-DEXK phosphodiesterase superfamily, suggesting that all Cas4 proteins are active nucleases. All members of the Cas4 family contain a Fe-S-cluster-binding module with 4 conserved cysteines. It has been shown that different Cas4 protein bind either [2Fe-2S] or [4Fe-4S] clusters [2, 15]; however, no consistent difference in the patterns of sequence conservation was detected between proteins binding these distinct ligands.

**Fig. 1:**
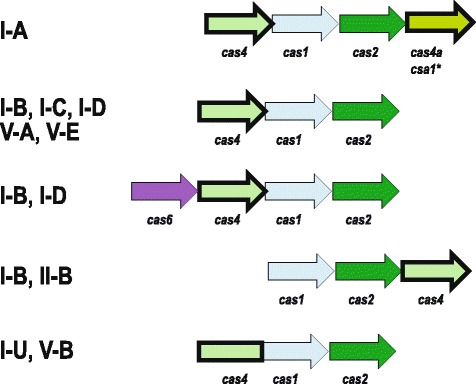
Typical organization of adaptation modules that include *cas4* genes. Most common organizations of adaptation modules containing *cas4* gene are shown. Genes are shown by block arrows according to the transcription direction and are not to scale. Homologous genes are color-coded and identified by a systematic name and a legacy name (indicated by an asterisk). Cas4 family genes are indicated by thick outline. CRISPR-Cas system subtypes found in association with each module are indicated. Notably, the adaptation modules of the I-B and I-D loci include *cas6* genes

We then constructed a sequence similarity dendrogram (see Materials and Method for details) for the 7060 identified Cas4 family proteins and mapped the gene context to each branch, including assignments to CRISPR-Cas subtypes (Additional file 1: Figure S1). As expected, the sequences that belong to distinct pfam entries and COGs do not overlap in the tree, and pfam12705 contained no major branches associated with CRISPR-Cas systems; thus, hereinafter, we only briefly discuss this family. The remaining Cas4 homologs could be classified into three major groups based on the genomic context: 1) CRISPR-Cas-associated Cas4 (CAS-Cas4), 2) Cas4 associated with mobile genetic elements (MGE-Cas4), and 3) solo-Cas4 (i.e. *cas4* genes located outside functionally characterized genomic contexts). The CAS-*cas4* genes are embedded in several typical arrangements of the adaptation module that also include the *cas1* and *cas2* genes, and are often shared between several CRISPR-Cas subtypes (Fig. 1). As reported previously, Cas1 and Cas4 proteins are fused in some systems (I-B, I-U and V-A), whereas I-A is the only CRISPR-Cas subtype that typically contains two *cas4*-*like* genes from two distinct subfamilies within the adaptation module (Fig. 1). One of the conserved arrangements shared by some of I-B and I-D systems also includes the *cas6* gene, which is not known to be involved in adaptation, but has been identified in several fusions with Cas1 and reverse transcriptase domains in some type III CRISPR-Cas systems (Fig. 1) [29, 30].

**Fig. 2:**
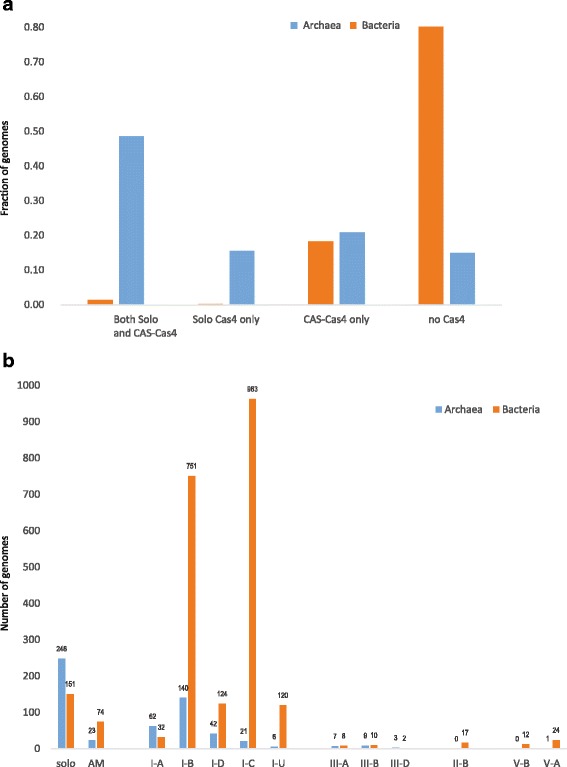
Representation of different Cas4 groups in archaea and bacteria. **a**. Presence and absence of CAS-Cas4 and Cas4 solo in completely sequenced genomes of archaea and bacteria. Individual genomes were assigned weights inversely proportional to the number of genomes from each species; the presence fraction was calculated with genome weights taken into account (see Additional file 4: File S2). **b**. Numbers of species in the complete data set containing Cas4 from different CRISPR-Cas4 subtypes and Cas4 solo. The counts were obtained from the complete set of CAS-Cas4 and Cas4-solo (5571 and 702, respectively). Only one strain per species was counted for each respective category of Cas4 assignments to avoid sequencing bias. See Additional file 7: Table S1 for details. Abbreviation: AM, stand-alone adaptation module

We further compared the phyletic distributions of the CAS-Cas4 and solo-Cas4 in complete microbial genomes (Fig. 2a). This analysis revealed pronounced differences between the distributions of the Cas4 family members in archaea and bacteria. The Cas4 family is represented in about 90% of the archaeal genomes, and about 50% of these encode both CAS-Cas4 and solo-Cas4 (Additional file 4: Information File S2). We further compared the representation of solo-Cas4 and distinct CAS-Cas4 contexts for the complete genome set, including draft genomes, counting one strain per species for each category to avoid sequencing bias (Fig. 2b). Bacteria show a patchy distribution of the Cas4 family genes, with a smaller fraction of solo-Cas4 compared to archaea (Fig. 2b). Numerous archaeal genomes encode both CAS-Cas4 and solo-Cas4 but the frequency of this combination is close to the product of individual frequencies of each form, thus showing no evidence of a functional connection. Notably, most members of the DPANN superphylum (a recently discovered major, apparently monophyletic group of archaea that includes *Aenigmarchaeota, Diapherotrites, Micrarchaeota, Nanohaloarchaeota, Pacearchaeota, Parvarchaeota*, and *Woesearchaeota*), largely symbiotic archaea with small genomes [31, 32], encode solo-Cas4, but not CAS-Cas4 (Additional file 7: Table S1). Within the entire genomic set, which includes draft genomes, CAS-Cas4 is most often present in type I-B and I-C CRISPR-Cas loci (Fig. 2b). We also observed many cases of CAS-Cas4 being a component of stand-alone (not associated with effector modules) adaptation modules, which are present in about 12% of the archaea and 4% of the bacteria that possess at least one *cas4* gene (Fig. 2b). Conceivably, the high prevalence of Cas4 in archaea is due to the dominance of I-B and I-C systems as opposed to I-E and I-F that are most common in bacteria and lack the *cas4* gene [11]. The biological underpinnings of this distinct distribution of Cas4-containing CRISPR-Cas remains obscure.

**Fig. 3:**
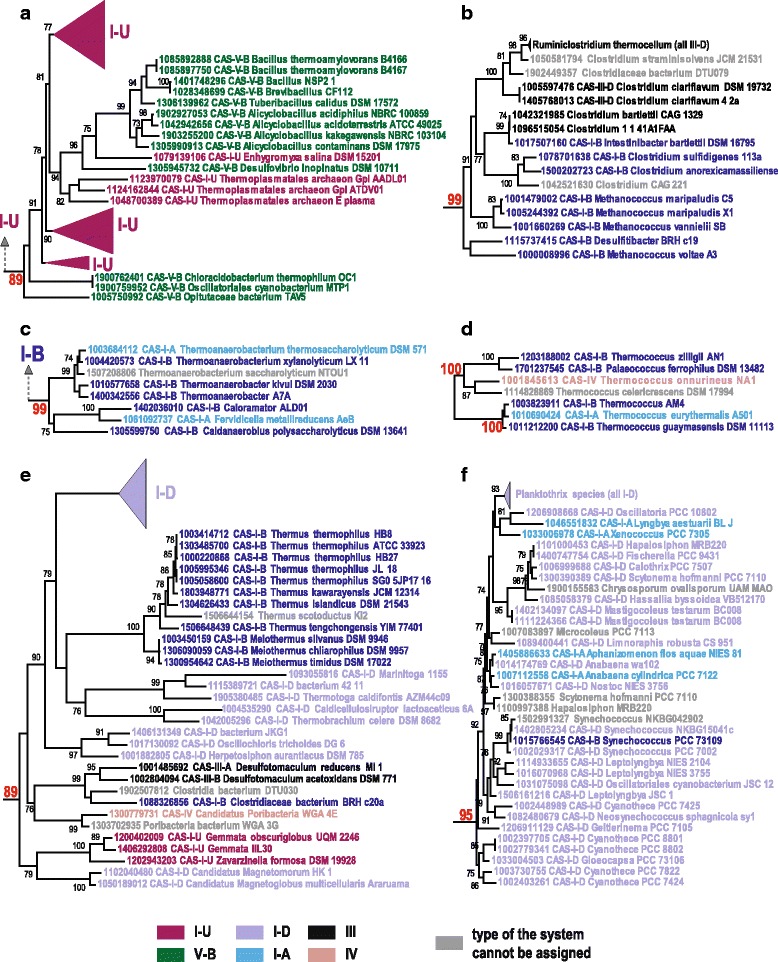
Selected examples of confidently supported subtrees of Cas4 associated with different subtypes of CRISPR-Cas effector complexes. Strongly supported (support values indicated in red) subtrees correspond to branches from the complete Cas4 tree shown in Additional file 1: Figure S1 and available in Newick format as Supplementary Information File 1.Each sequence is denoted by a numeric identifier, matching those in Additional file 7: Table S1, subtype of the CRISPR-Cas system from the respective locus and species name. Several branches were collapsed and are indicated by triangles with CRISPR-Cas system subtype indicated next to respective triangle. The coloring of the sequences corresponds to distinct CRISPR-Cas system types or subtypes according to the color code provided at the bottom

Phylogenetic analysis indicates that the majority of the CAS-Cas4 proteins form clades that are compatible with the classification of the CRISPR-Cas subtypes delineated through comparison of effector gene sets. However, there are many exceptions to this congruence, so that, in the phylogenetic tree of Cas4, only subtypes I-C, II-B, and V-A are both monophyletic and compact (i.e. do not include representatives of other subtypes) (Fig. 3, Additional file 1: Figure S1, and Additional file 3: Information File S1). In agreement with the previous phylogenetic analysis of Cas1, the subtype II-B-associated Cas4 is nested within the I-B/I-A branch, whereas the subtype V-B-associated Cas4 proteins, although not forming a clade, fall within the I-U branch (Additional file 1: Figure S1, Additional file 3: Information File S1) [33] (Fig. 3a, Additional file 1: Figure S1, Additional file 3: Information File S1). Thus, the Cas4-containing adaptation modules in subtypes II-B and V-B apparently were acquired from the respective type I systems. In contrast, the subtype V-A-associated Cas4 is distinct and does not show clear affinity to any known CRISPR-Cas systems types (Additional file 1: Figure S1, Additional file 3: Information File S1). This observation is in agreement with the long, deep V-A branch in the respective Cas1 phylogeny [33].

Subtypes I-A, I-B, I-D and to a lesser extent I-U, are scattered among many strongly supported Cas4 branches suggesting extensive shuffling of the adaptation and effector modules (Fig. 3b-f, Additional file 1: Figure S1, Additional file 3: Information File S1). This evidence of module shuffling is consistent with previous observations from phylogenetic analysis of Cas1, although this effect has been underestimated and was not carefully explored. With many more genomes now available for analysis, it becomes apparent that adaptation modules of subtypes I-A, I-B and I-D can function with (almost) any of the respective effector modules (Fig. 3, Additional file 1: Figure S1, Additional file 3: Information File S1). Furthermore, these adaptation modules apparently can combine also with type III and type IV effector modules (Fig. 3, Additional file 1: Figure S1, Additional file 3: Information File S1) because some branches within well-supported Cas4 clades correspond to these CRISPR-Cas types (Fig. 3, Additional file 1: Figure S1, Additional file 3: Information File S1).

**Fig. 4:**
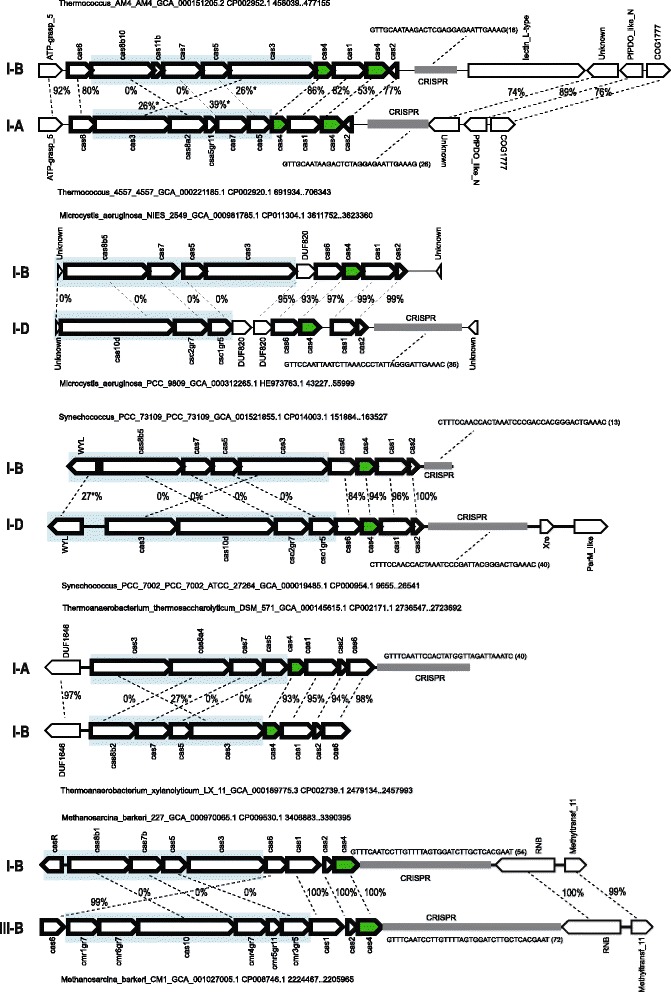
Selected examples of in situ replacement of effector complex genes in CRISPR-Cas systems of different subtypes. For each locus, species name, genome accession number and the respective nucleotide coordinates are indicated. The genes of a representative locus are shown by arrows. The arrow indicates the transcription direction of the respective gene. Genes and CRISPR arrays are shown roughly to scale. Homologous genes are connected by dotted lines and amino acid sequence identity (%) is indicated. Asterisks indicate cases where the overall sequence identity was very low, and the percent identity was taken from a small aligned fragment. Cas4 genes are shown in green. The region of likely in situ replacement is shown by a blue rectangle

**Fig. 5:**
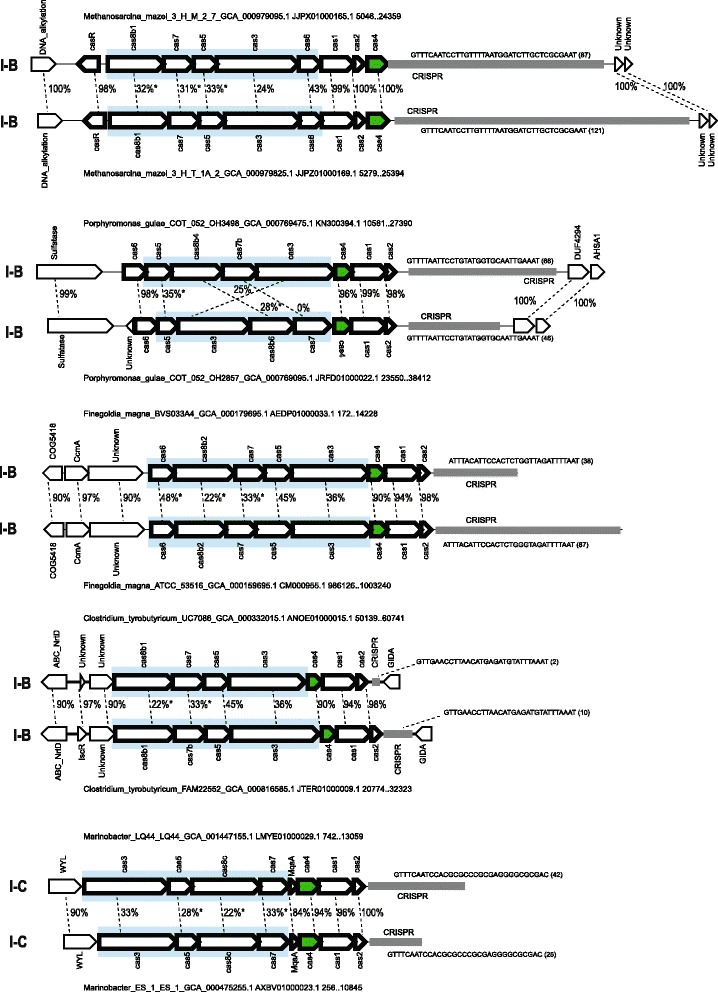
Selected examples of in situ replacement of effector complex genes in CRISPR-Cas loci of the same subtype. Designations are the same as in the Fig. 4

We examined in greater detail several cases where highly similar *cas4* genes were associated with different CRISPR-Cas system (sub)types, especially in closely related species or strains, where *cas4* genes could be expected to be vertically inherited (Fig. 4). Analysis of these cases suggests that adaptation module genes remain untouched by recombination, whereas effector genes replace the ancestral effector modules. This inference of the preferred directionality of recombination events is supported, in particular, by the presence of flanking syntenic regions (Fig. 4). These observations prompted us to search for similar exchanges within the same CRISPR-Cas subtype. To this end, we clustered the large subunits of the respective systems (Cas8 for type I-A, I-U and I-B and Cas10d for I-D) with permissive cutoffs and mapped these clusters on the tree in order to identify cases where closely related *cas4* genes are co-located with substantially different groups of large subunits of the same subtype. This procedure allowed us to identify additional cases where effector module genes are replaced, whereas the adaptation module remained unaffected (Fig. 5). In many of these cases, flanking syntenic regions allow identification of the precise boundaries of the effector gene replacement sequences, even on the nucleotide level (Additional file 2: Figure S2). We also noticed some cases when the effector genes are deleted, resulting in emergence of stand-alone adaptation modules that are usually located inside the branch for the respective subtypes and were identified in many archaea and bacteria (Fig. 2b, Additional file 1: Figure S1, Additional file 3: Information File S1).

Solo-Cas4 can be clearly differentiated from CAS-Cas4 in the phylogenetic tree where they form several distinct branches that are mostly consistent with the order-level taxonomy of archaea, suggesting predominant vertical inheritance (Additional file 1: Figure S1, Additional file 3: Information File S1). Despite the general trend to retain solo-Cas4 in the genome, in some archaeal lineages, this gene apparently evolved fast, so that, for example, two groups of *Thermoproteales, Pyrobaculum/Thermoproteus* and *Vulcanisaeta*, encode dissimilar solo-Cas4 sequences that do not cluster with each other or with homologs from other archaea. Several members of the archaeal DPANN superphylum that mostly lack CRISPR-Cas systems, encode two distinct solo-Cas4 proteins (Additional file 1: Figure S1, Additional file 3: Information File S1). In bacteria, solo-Cas4 shows a patchy distribution, with some notable exceptions. First, solo-Cas4 is present and monophyletic in the majority of *Thermoanaerobacteriales* and many *Chloroflexi.* Second, solo-Cas4 is encoded in many genomes of the Candidate Phyla Radiation (CPR), a recently described assemblage of bacteria with small genomes and many unusual features, such as *Parcubacteria, Nomurabacteria, Roizmanbacteria* and others [34, 35]. Some of the solo-Cas4 proteins from the CPR confidently group with homologs from the archaeal DPANN superphylum (Additional file 1: Figure S1, Additional file 3: Information File S1). Thus, solo-Cas4 is clearly over-represented in environmental bacteria and archaea with small genomes and could perform important biological functions in these organisms that remain to be characterized. Furthermore, dissemination of solo-Cas4 genes via HGT between bacteria and archaea appears likely. In several of the DPANN archaeal genomes, the gene context, namely the adjacency to genes coding for restriction-modification systems, implies that solo-Cas4 functions in so far uncharacterized defense pathways (Additional file 7: Table S1).

### Cas4 in mobile genetic elements (MGE-Cas4)

Phylogenetic analysis of the Cas4 family identified several distinct branches that are associated with MGE and viruses, especially, archaeal ones; to our knowledge, the spread of Cas4 homologs in MGE has not been systematically described previously. In particular, a *cas4* gene is present in most of the known viruses of Desulfurococcales and Sulfolobales (Additional file 1: Figure S1, Additional file 3: Information File S1). For example, Cas4 proteins encoded in Acidianus filamentous virus genomes form a lineage that is the sister group of solo-Cas4 from *Sulfolobales* within the archaeal solo-Cas4 branch, suggesting acquisition of these genes from the host by the ancestral virus (Additional file 1: Figure S1, Additional file 3: Information File S1). Sulfolobus spindle shaped virus encodes Cas4 that is most similar to a group of Cas4 proteins associated with type I CRISPR-Cas systems in *Thermococcales* (Additional file 1: Figure S1, Additional file 3: Information File S1). In this case, the ancestral virus seems to have captured a CAS-Cas4 that became solo in the virus genome. Several Cas4 proteins from Sulfolobus islandicus rudiviruses and Acidianus filamentous virus also form a branch, albeit weakly supported. Cas4 proteins from numerous phages form a large, well-supported clade which includes many phages of Cyanobacteria and Proteobacteria, suggesting dissemination of the *cas4* gene among phages. One branch in the Cas4 tree cannot be linked to any known phages but likely corresponds to uncharacterized prophages integrated in genomes of *Campylobacteriales.* Another large clade of Cas4 is apparently associated with conjugative plasmids from *Burkholderiales*. Other Cas4 proteins encoded by bacteriophages and archaeal viruses are scattered over the tree and typically do not show clear affinity to solo-Cas4, CAS-Cas4 or each other (Additional file 1: Figure S1, Additional file 3: Information File S1).

### Horizontal gene transfer and selection pressures on different groups of *cas4* genes.

To estimate the relative contributions of the vertical and horizontal components in the evolutionary history of the *cas4* gene family, we compared the tree-induced distances between Cas4 protein sequences and the distances between the 16S rRNAs from the corresponding genomes (proxy for the species tree) as described earlier [11] (see Methods for details); a parallel analysis of Cas1 proteins was used as a reference (see Methods for the link to the data available online).

Both Cas4 and Cas1 only loosely follow the species tree in their history: the Spearman rank correlation coefficients with the 16S rRNA distances for CRISPR-Cas associated Cas4 and Cas1 are 0.15 and 0.22, respectively (see Methods for details). Notably, both solo *cas4* genes and solo *cas1* genes show a stronger vertical evolution trend (correlation coefficients of 0.47 and 0.53, respectively). The substantially greater horizontal component in the evolutionary history of the CRISPR-associated genes reflects the more dynamic nature of the evolution of defense systems compared to other categories of microbial genes [36]. The *cas4* and *cas1* genes encoded in the same locus nearly always evolve (and get transferred) as a single unit (see Methods for the link to the data available online): the correlation between the *cas4* and *cas1* distances for co-located genes is 0.88, and both show closely similar, low correlation coefficients with the rRNA tree (0.14 and 0.13, respectively).

All *cas4* genes seem to evolve under comparable levels of selective pressure, with the medians of the *dN/dS* ratios for the CAS-cas4 and solo-cas4 gene pairs being in the range of 0.06 to 0.18 (Additional file 5: Information file S3). These values are close to the genome-wide median *dN/dS* ratios that are in the range of 0.10 to 0.14 [37]. Thus, in agreement with previous observations [38], all *cas4* genes experience moderate levels of purifying selection that are close to the median values for the respective bacteria and archaea. Furthermore, it has been shown that the characteristic selective pressure on *cas4* gene is only slightly lower than that on *cas1* and *cas2*, the most strongly conserved *cas* genes, which is compatible with the joint involvement of the respective Cas proteins in adaptation [38].

### Cas4-like proteins of pfam12705

As mentioned above, Cas4-like proteins that showed the highest similarity to pfam12705 are not associated with CRISPR-Cas systems. However, in addition to the *Campylobacter* phages that encode a Cas4-like nuclease that has been shown to interfere with the host type II-C CRISPR-Cas system [22, 23], members of this family were identified in several other notable contexts. These include a large clade that consists of phages infecting different *Mycobacteria* as well as some *Propionibacterium* and *Bacillus* phages as well as several smaller branches including phages that infect *Bacillus*, *Thermus* and other bacteria (Additional file 1: Figure S1, Additional file 3: Information File S1).

**Fig. 6:**
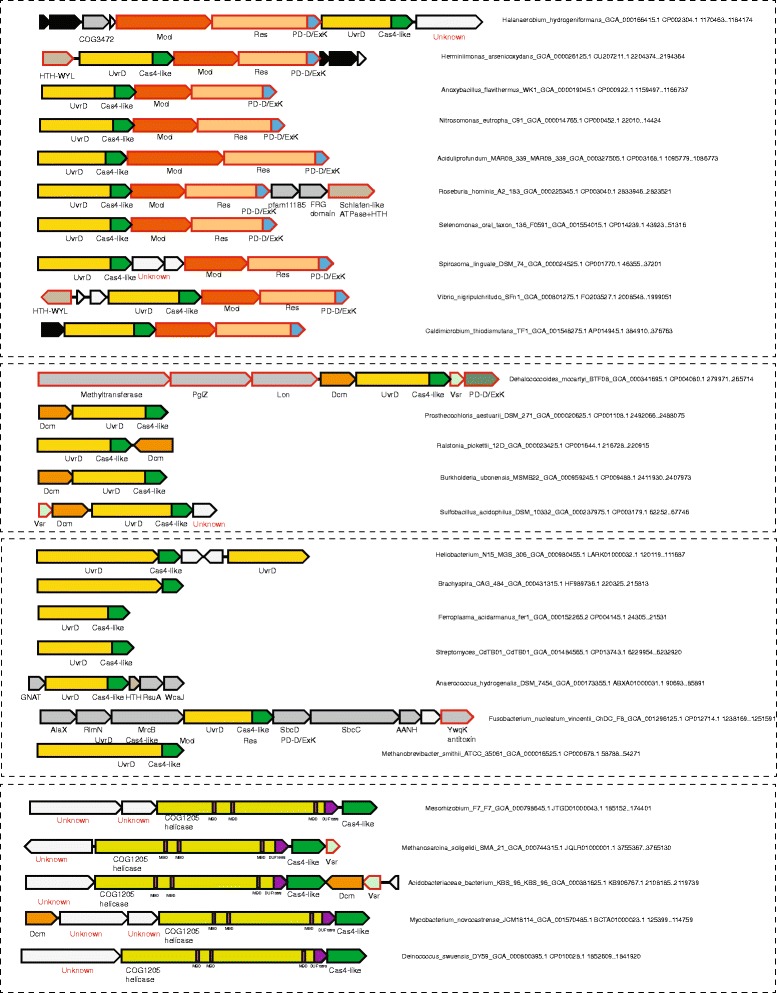
Organization of loci containing UvrD-like, COG1205 and Cas4-like genes. For each locus, species name, genome accession number and the respective nucleotide coordinates are indicated. The genes of a representative locus are shown by arrows. The scale of an arrow is roughly proportional to gene length. The arrow indicates direction of the respective gene. Homologous genes and domains are color-coded. Four panels separated by dotted rectangles show a common theme of locus organization. Abbreviations and gene names: Mod –modification subunit of type III RM system, Res – restriction enzyme of type III RM system; Dcm - DNA-cytosine methylase; HTH- helix turn helix; Vsr – very short patch repair nuclease, PD-DExK – PD-DExK family nuclease; WcaJ-Sugar transferase involved in LPS biosynthesis; RsuA - Pseudouridine synthase; MrcB - Membrane carboxypeptidase; RlmN - Adenine C2-methylase of 23S rRNA A2503 and tRNA A37; AlaX - Ser-tRNA(Ala) deacylase; AANH - Adenine nucleotide alpha hydrolases

The largest branch in the pfam12705 family includes proteins from many bacteria and mesophilic archaea, in which a Cas4-like nuclease domain is fused to a UvrD-like superfamily 1 (SF1) 3′-5′ helicase (Fig. 6). Fusion of RecB family nucleases with SF1 helicases is common, e.g. in RecB and AddB, key proteins in bacterial recombinational repair pathways [39]. The UvrD helicase has been originally identified as a component of the nucleotide excision repair complex along with UvrA and UvrB proteins, but subsequently, has been shown to participate in the regulation of RecA recombinase, mismatch repair, transcription-coupled repair, and chromosome and plasmid replication, as an accessory helicase [39, 40]. Many bacteria encode multiple UvrD (COG0210) paralogs [41].

We constructed a tree of UvrD homologs from all genomes that encode the UvrD-Cas4-like fusion and found that the helicase domains of the fusion proteins are also monophyletic, indicating a single origin and, most likely, a unique function of the fusion proteins (Additional file 6: Information File S4). The UvrD-Cas4 proteins are encoded in two distinct, conserved contexts. The most common genomic neighborhood includes genes for methylation (Mod) and restriction (Res) subunits of Type III Restriction-Modification systems [42–44]. Several of these loci also encompass genes for predicted regulatory proteins containing WYL and helix-turn-helix (HTH) domains as well as Shlaffen-like ATPases fused to an HTH domain that are often encoded in other defense contexts.

Another context of the UvrD-Cas4 fusion includes DNA-cytosine methylase Dcm that is responsible for all 5-methylcytosine modifications in *E. coli* [45]. The physiological role of this methylation remains unknown although it has been shown that Dcm recognition sites correspond to sequence motifs for very short patch repair of T/G mismatches, and the Vsr nucleases involved in this process are often encoded next to the *dcm* gene [45]. Indeed, several *uvrD-cas4* loci also contain a gene for a Vsr nuclease, suggesting that this system retains the same features of recognition sites and functional link between Dcm and Vsr (Fig. 6). The same pair of genes was observed previously in a variety of defense gene contexts, MORC family ATPases in particular [46]. Collectively, these genomic associations strongly suggest that the UvrD-Cas4 helicase-nuclease fusion proteins are components of multiple microbial defense systems.

Considering that, in both cases described above, the UvrD-Cas4 protein appears to belong to the same subfamily and the *uvrD-cas4* gene is either stand-alone or is found in non-conserved contexts in archaeal and bacterial genomes, it appears that the fusion protein plays an ancillary role in different pathways by facilitating site-specific DNA unwinding and cleavage. The Cas4-like nuclease domain can be predicted to play a role analogous to the nuclease domains of RecB and AddB in promoting DNA recombination pathways [19]. Most of the bacterial genomes that encode the UvrD-Cas4 fusion also encode RecB (a near ubiquitous protein in bacteria) suggesting that the functions of these helicase-nuclease fusion proteins could be partially redundant rather than complementary, a pattern typical of repair pathways [47, 48].

The link between UvrD and Cas4-like protein evolved at least on one other independent occasion. In several bacteria, mostly Clostridiales, the two genes are not fused but are encoded in a predicted operon in a context that does not appear indicative of defense functions, suggesting that the two enzymes could be jointly involved in other cellular processes, such as DNA repair (Fig. 6).

Finally, in several bacteria and mesophilic archaea, we identified another link between a Cas4-like nuclease and a superfamily 2 helicase that belongs to the uncharacterized YprA-like family (COG1205) and is unrelated to UvrD. This helicase contains a characteristic C-terminal metal-binding domain (pfam09369/DUF1998 family) and large inserts with additional putative metal-binding domains (Fig. 6). Helicases of this family are strongly associated with defense islands [49], suggesting that, together with the Cas4-like protein, they participate in so far uncharacterized defense pathways.

## Conclusions

Unlike most *cas* genes, Cas4 family endonucleases are about as often encoded outside as they are within CRISPR-*cas* loci and are also found in many MGE, particularly archaeal viruses. Because of this unusual distribution of *cas4* genes in bacterial and archaeal genomes, we sought to explore the phylogenomics of the Cas4 family. The results of this phylogenomic indicate that evolution of those *cas4* genes that belong to CRISPR-Cas adaptation modules (Cas-*cas4*) involves extensive horizontal transfer that, however, almost always involves complete adaptation modules. The evolutionary integrity of the adaption modules sharply contrasts the rampant modular shuffling of CRISPR-Cas systems whereby a given variant of the adaptation module can combine with virtually any variety of effector modules. The evolutionary history of solo-*cas4* genes is much better compatible with the microbial species tree and appears to include fewer horizontal gene transfer events. The Cas-*cas4* and solo-*cas4* genes evolve under moderately strong purifying selection that, for both classes of *cas4* genes, is close to the genomic median. Thus, the evolutionary regime of the *cas4* genes, along with *cas1* and *cas2* genes, resembles that of ‘regular’ microbial genes involved in various cellular functions, with no evidence of direct involvement in virus-host arms races. However, *cas4* genes were recruited by various MGE on many independent occasions, suggesting that Cas4 nucleases might be involved in anti-defense functions. The solo-Cas4 as well as MGE-encoded Cas4 so far have not been studied experimentally. Functional characterization of these proteins is likely to result in identification of novel defense and anti-defense systems, and their connections with CRISPR-Cas systems.

## Methods

### Data

The prokaryotic genomic dataset was taken from WGS and GenBank repositories at the NCBI as of March 2016 and includes 43,000 genomes. Cas4-like proteins were identified using PSI-BLAST search against sequence profiles corresponding to the following subfamilies: COG1468, COG4343, pfam01930, pfam06023, cd09637, cd09659, cls000170 [11] and pfam12705. To ensure specificity of the extracted set of proteins after the initial identification, all retrieved protein sequences were additionally run against the Conserved Domain Database (CDD) using the RPS-BLAST program with the same parameters and all proteins with higher similarity to any other profiles were discarded. The protein sequences from the *cas4* neighborhoods were extracted from the respective genomes or contigs and annotated using the COGs, Pfam and CD profiles from the CDD and the custom defense gene profiles [11]. The *cas* genes and CRISPR-Cas subtypes were annotated according to the previously described nomenclature [11].

### Clustering and Phylogenetic analysis

The NCBI BLASTCLUST program (ftp://ftp.ncbi.nih.gov/blast/documents/blastclust.html) was used to cluster sequences by similarity. For Cas4 clustering, BLASTCLUST was used with the sequence identity threshold of 50% and length coverage threshold of 0.8; for 16S rRNA nucleotide sequences, the program was used with sequence identity threshold of 90% and length coverage threshold of 0.9. For Cas8 and Cas10d clustering, the program was used with sequence identity threshold of 0.5 and length coverage threshold of 80%. Alignments of protein sequences were constructed using MUSCLE [50] and MAFFT [51] programs. Short fragments or disrupted sequences were discarded. Sites with the gap character fraction values >0.5 and homogeneity <0.1 were removed from the alignment. Phylogenetic analysis was performed using the FastTree program [52], with the WAG evolutionary model for amino acid sequences and the GTR evolutionary model for nucleotide sequences. The same program was used to compute bootstrap values.

Relationships within diverse sequence families were established using the following procedure: initial sequence clusters were obtained using UCLUST [53] with the sequence similarity threshold of 0.5; sequences were aligned within clusters using MUSCLE [50]. Then cluster-to-cluster similarity scores were obtained using HHsearch [54] (including trivial clusters consisting of a single sequence each); a UPGMA dendrogram was constructed from the pairwise similarity scores. Highly similar clusters (pairwise score to self-score ratio > 0.1) were aligned to each other using HHALIGN [55]; the procedure was repeated iteratively. At the last step, sequence-based trees were reconstructed from the cluster alignments using FastTree [52], as described above and rooted by mid-point; these trees were grafted onto the tips of the profile similarity-based UPGMA dendrogram.

For the comparison of evolutionary distances between Cas4 and Cas1 protein sequences and 16S rRNA sequences from the corresponding genomes (proxy of the species tree), all pairwise distances between CRISPR-associated or solo proteins were calculated from the corresponding phylogenetic trees; for protein sequences from unaligned clusters, an arbitrary high distance of 12 was assigned to the corresponding pair. If a pair of genomes contained more than one Cas4 or Cas1 sequence in either or both of them, the shortest protein-protein distance was selected to represent this genome pair. Distances between the corresponding 16S rRNA sequences were, likewise, calculated from the 16S rRNA trees or, if the two 16S rRNAs belonged to unaligned clusters, an arbitrary distance high distance of 3 was assigned. Spearman rank correlation between protein-protein and rRNA-rRNA sequence distances for all pairs was reported as a measure of coherence between the protein evolution and the species tree.

All pairwise distances are available for download at ftp://ftp.ncbi.nih.gov/pub/wolf/_suppl/cas4.

## Additional files

Additional file 1: Figure S1. Schematic representation of the maximum likelihood phylogenetic tree of Cas4 (7060 sequences all together), available in the Supplementary File 1. Support values are calculated by FastTRee program only for the confidently aligned groups, all other values were assigned to zero automatically. Major well-supported distinct branches are shown by rectangles which are color-coded according to Cas4 assignments. Assignments and other comments are shown next to the each collapsed branch. Individual sequences in the tree are described by a local numeric ID, species name and color-coded according to Cas4 assignment (also provided in the Additional file 7: Table S1). Blue shading shows tree clades that belong to pfam12705 family. (PDF 694 kb)

Additional file 2: Figure S2. Nucleotide sequence comparisons of CRISPR-Cas loci encoded in closely related strains. On the axes, the labels contain the name of the source genome, contig ID and the coordinates of the respective loci. The annotations for CRISPR-Cas loci were taken from the Additional file 7: Table S1, “Loci” worksheet. The cartoons on the axes represent the genes and CRISPR repeats encoded in these loci. The sizes of the cartoons are proportional to the actual sizes of these genes. Colors: black are CRISPR arrays, blue are cas genes, green - cas4 gene, shaded area are the regions which have >70% sequence identity level. Left: Comparison of two I-C systems from Marinobacter strains. Right: Comparison of two I-B systems from Campylobacter strains. (DOCX 606 kb)

Additional file 3: Information File S1. Complete tree for Cas4-like set in Newick format. Cas4 assignments are included in the leaf name. (TXT 463 kb)

Additional file 4: Information File S2. Breakdown of CAS-Cas4 and solo-Cas4 presence in completely sequenced genomes. (XLSX 248 kb)

Additional file 5: Information File S3. Detailed description of the Cas4 *dN/dS* analysis, table with results. (DOCX 51 kb)

Additional file 6: Information File S4. Complete tree for selected representatives of UvrD family in Newick format. The tree is based on the helicase domain alignment. Protein linked to Cas4 are indicated in the respective leaf names. (DOCX 51 kb)

Additional file 7: Table S1 Worksheet “loci” provides detailed information on all cas4 loci in completely sequenced and draft genomes of archaea and bacteria. Annotation for the proteins encoded in the loci is based on Cas protein and CDD assignments using PSI-BLAST program (see Methods for details). Worksheet “tree order and assignments” provides information of the order of the Cas4 in the tree (Supplementary file 1), Cas4 assignments to distinct groups of CAS-Cas4, solo-Cas4 and others. (TXT 28 kb)
