# Supplementary figures and images for: Phylogenomics of Cas4 family nucleases
Source: BMC Evol Biol. 2017 Nov 28;17:232. doi: 10.1186/s12862-017-1081-1 (PMC5704561; doi:10.1186/s12862-017-1081-1)

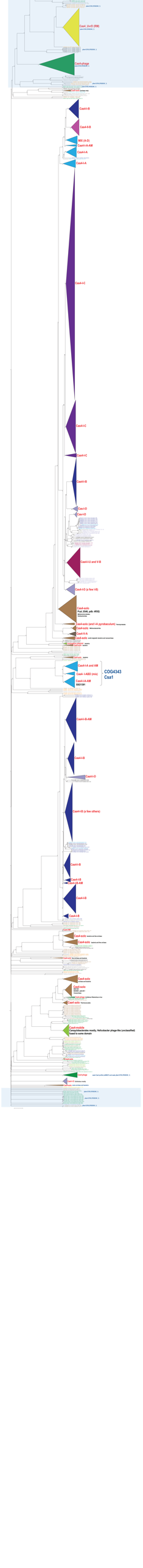

Supplement: Supplementary file 1 — Schematic representation of the maximum likelihood phylogenetic tree of Cas4 (7060 sequences all together), available in the Supplementary File 1. Support values are calculated by FastTRee program only for the confidently aligned groups, all other values were assigned to zero automatically. Major well-supported distinct branches are shown by rectangles which are color-coded according to Cas4 assignments. Assignments and other comments are shown next to the each collapsed branch. Individual sequences in the tree are described by a local numeric ID, species name and color-coded according to Cas4 assignment (also provided in the Additional file 7: Table S1). Blue shading shows tree clades that belong to pfam12705 family. (PDF 694 kb) [file 12862_2017_1081_MOESM1_ESM.pdf]
