# Supplementary material for: Phylogenomics of Cas4 family nucleases
Source: BMC Evol Biol. 2017 Nov 28;17:232. doi: 10.1186/s12862-017-1081-1 (PMC5704561; doi:10.1186/s12862-017-1081-1)
Supplement: Supplementary file 2 — Nucleotide sequence comparisons of CRISPR-Cas loci encoded in closely related strains. On the axes, the labels contain the name of the source genome, contig ID and the coordinates of the respective loci. The annotations for CRISPR-Cas loci were taken from the Additional file 7: Table S1, “Loci” worksheet. The cartoons on the axes represent the genes and CRISPR repeats encoded in these loci. The sizes of the cartoons are proportional to the actual sizes of these genes. Colors: black are CRISPR arrays, blue are cas genes, green - cas4 gene, shaded area are the regions which have >70% sequence identity level. Left: Comparison of two I-C systems from Marinobacter strains. Right: Comparison of two I-B systems from Campylobacter strains. (DOCX 606 kb) [file 12862_2017_1081_MOESM2_ESM.docx]

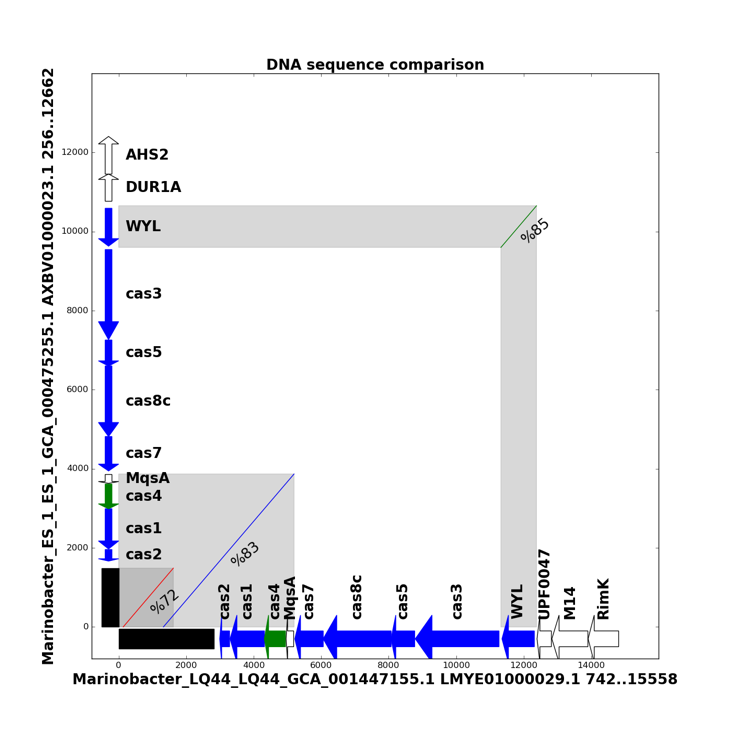

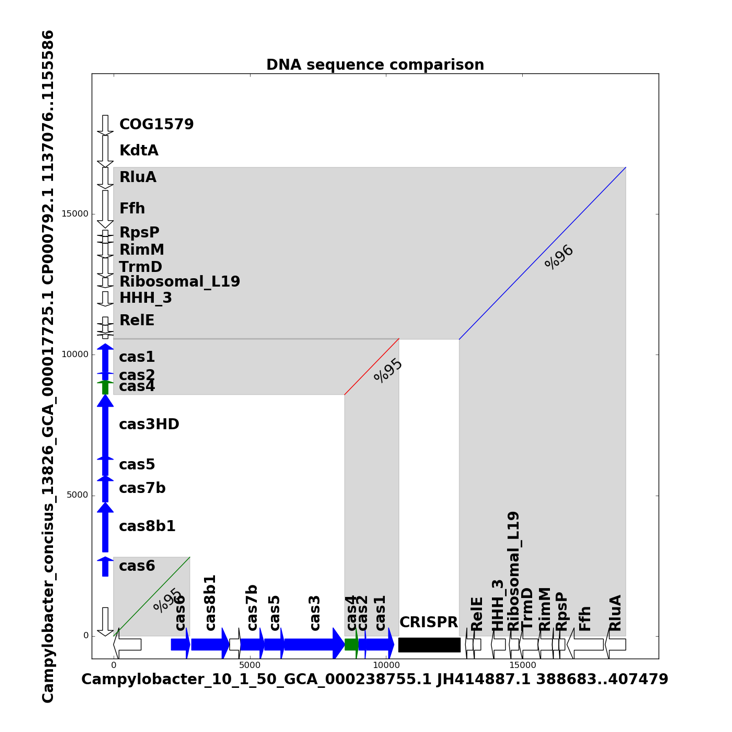


**Supplementary Figure 2**. Nucleotide sequence comparisons of CRISPR-Cas loci encoded in closely related strains. On the axes, the labels contain the name of the source genome, contig ID and the coordinates of the respective loci. The annotations for CRISPR-Cas loci were taken from the Supplementary Table 1, “Loci” worksheet. The cartoons on the axes represent the genes and CRISPR repeats encoded in these loci. The sizes of the cartoons are proportional to the actual sizes of these genes. Colors: *black* are CRISPR arrays, *blue* are *cas* genes, *green* - *cas4* gene, *shaded area* are the regions which have >70% sequence identity level. **Left**: Comparison of two I-C systems from *Marinobacter* strains. **Right**: Comparison of two I-B systems from *Campylobacter* strains.
