# Supplementary material for: Phylogenomics of Cas4 family nucleases
Source: BMC Evol Biol. 2017 Nov 28;17:232. doi: 10.1186/s12862-017-1081-1 (PMC5704561; doi:10.1186/s12862-017-1081-1)
Supplement: Supplementary file 5 — Detailed description of the Cas4 dN/dS analysis, table with results. (DOCX 51 kb) [file 12862_2017_1081_MOESM5_ESM.docx]

**Analysis of evolutionary constraints**

To estimate the selective pressure on different classes of Cas4 proteins, the following procedure was used (Figure 1). All identified Cas4 sequences were classified into categories according to their predicted and genomic context (CAS-associated Cas4, solo Cas4, etc.) as described in details in the main text. For each of the classes, the following steps were performed:

1. Cluster the sequences with the identity threshold level of %60 (UCLUST).
2. Make a multiple alignment from each of the clusters (MUSCLE).
3. Construct a phylogenetic tree from each of the alignments (FastTree).
4. From each of the trees, parse out the sub-trees with the depth threshold level of $d=0.1$ from the leaves.
5. Re-align the leaf sequences from the sub-trees, retrieve the corresponding DNA sequences and generate codon alignments, based on the multiple alignment of the corresponding proteins.
6. Calculate $dN$, $dS$ and $dN/dS$ values for al pairs of aligned sequences (PAML/codeml).
7. Select the pairs with $dN$ and $dS$ values within the range of [0.0002, 1].

The distributions of $dN$/$dS$ values for all categories are summarized in the Table 1.


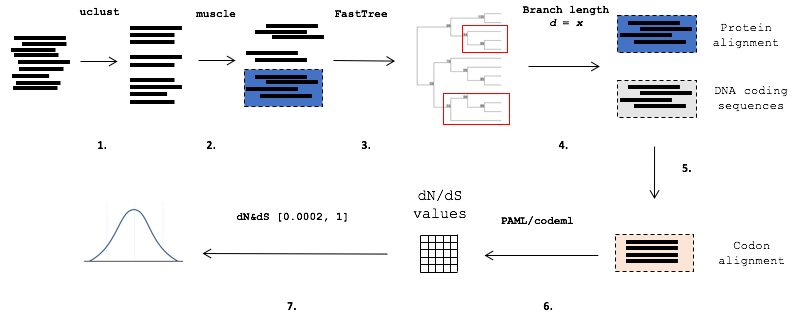


**Figure 1.** Workflow of calculating $dN/dS$ values in *cas4* genes. The steps are described above.

|  |  |  |  | $\boldsymbol{dN/dS}$ | | |
| --- | --- | --- | --- | --- | --- | --- |
| **Category** | | **Genes** | **Pairs** | **1st quartile** | **Median** | **4th quartile** |
| **Fusion** | **Cas4&UvrD** | 51 | 104 | 0.06 | 0.08 | 0.11 |
|  | **Cas4** | 51 | 43 | 0.02 | 0.06 | 0.12 |
|  | **UvrD** | 51 | 50 | 0.05 | 0.07 | 0.09 |
| **CRISPR loci** | **All** | 735 | 477 | 0.05 | 0.14 | 0.32 |
|  | **Complete** | 595 | 405 | 0.05 | 0.16 | 0.33 |
|  | **Incomplete** | 140 | 9 | 0.06 | 0.09 | 0.13 |
| **Solo** | | 803 | 795 | 0.08 | 0.18 | 0.49 |

**Table 1.** Average values of $dN/dS$ between pairs of sequences from different functional categories of *cas4* genes. Column *Pairs* denote number of genes passed through filtering process described above (Figure 1).
